# *Burkholderia cepacia* – outbreak in obstetric patients due to intrinsic contamination of non-sterile ultrasound gel

**DOI:** 10.1186/1753-6561-5-S6-O75

**Published:** 2011-06-29

**Authors:** M Hell, C Abel, A Albrecht, A Wojna, G Chmelizek, JM Kern, M Maass, P Apfalter

**Affiliations:** 1 Dep.of Hospital Epidemiology and Infection Control, University Hospital Salzburg, Salzburg, Austria; 2 Infection control team, Hallein, Austria; 3 Dep.of Gynecology , Teaching Hospital Hallein, Hallein, Austria; 4 Private Laboratories Dr.Mustafa/Dr.Richter, Dep.of clinical microbiology, University Hospital Salzburg, Salzburg, Austria; 5 Institute of medical microbiology, hygiene and infectious diseases, University Hospital Salzburg, Salzburg, Austria; 6 Institute of Hygiene, Medical Microbiology and Tropical Medicine, Teaching Hospital Elisabethinen, Linz, Austria

## Introduction / objectives

Ultrasound gel is a potential source of infection. Non-sterile ultra-sound gels can be contaminated due to manufacturing procedures and also during usage of opened bottles. We report a cluster of eight clinical cases of vaginal colonization (one clinically proven colpitis) with *Burkholderia cepacia* (B. cepacia) due to intrinsically contaminated ultrasound gel in obstetric patients in an Austrian hospital.

## Methods

When the cluster was realised a microbiological investigation of the environment was initiated (e.g. surfaces, equipment, ultrasound gels of different manufacturers). Isolates of three different patients and four isolates of ultrasound gel bottles (two opened and two sealed) were investigated by Puls-Field-Gel-Electrophoresis (PFGE) to clarify clonality and source.

## Results

Environmental specimens revealed no growth of B. cepacia. The four bottles from the incriminated manufacturer (two opened, two sealed bottles, all belonging to the same batch) were highly contaminated with B. cepacia (up to 40.000 CFU/ml). These isolates and the three patients' isolates showed the same genotype pattern by PFGE.

## Conclusion

We, therefore, concluded that the whole batch of these non-sterile gels was affected by monoclonal intrinsic contamination with B. cepacia due to insufficiently controlled manufacturing procedures. There is a need for discussion about microbiological contamination levels tolerated in such non-sterile ultrasound gels, especially when used on susceptible sites such as mucous membranes.

## Disclosure of interest

None declared.

